# The impact of omentectomy on cause-specific survival of Stage I–IIIA epithelial ovarian cancer: A PSM–IPTW analysis based on the SEER database

**DOI:** 10.3389/fsurg.2022.1052788

**Published:** 2022-12-29

**Authors:** Zhimin Hao, Yangli Yu, Sufen Yang

**Affiliations:** ^1^ Department of Gynecology, The Affiliated Hospital of Medical School of Ningbo University, Ningbo, China; ^2^ Medical School of Ningbo University, Ningbo, China

**Keywords:** epithelial ovarian cancer, omentectomy, lymphadenectomy, PSM, IPTW, regression tree model

## Abstract

**Objective:**

Routine omentectomy is generally performed during surgery for patients with epithelial ovarian cancer (EOC). The current study aims to evaluate the impact of omentectomy on cause-specific survival of Stage I–IIIA EOC patients.

**Methods:**

Patients who presented with clinical Stage I–IIIA serous, clear cell, endometrioid, and mucinous ovarian cancers were selected from the SEER Database for the period between 2004 and 2018. We extracted clinicopathological data and surgical information with the focus on the performance of omentectomy and lymphadenectomy. Binary logistic regression and recursive partitioning analyses were conducted to identify the significant factors for the performance of omentectomy during surgery. Propensity score matching (PSM) and inverse probability treatment weighting (IPTW) techniques were utilized to balance confounding factors. Multivariate, exploratory subgroup analyses and sensitivity analyses were conducted to evaluate the impact of omentectomy on cause-specific survival (CSS).

**Results:**

A total of 13,302 patients with EOC were enrolled in the study. The cohort comprised 3,569 endometrioid, 4,915 serous, 2,407 clear cell, and 2,411 mucinous subtypes. A total of 48.62% (6,467/13,302) of patients underwent the procedure of omentectomy during primary surgery, and only 3% absolute improvement in CSS at the individual level was observed, without statistical significance based on multivariate analysis. According to the regression-tree model with recursive partitioning analysis, the procedure of lymphadenectomy was found to be the strongest factor to distinguish the performance of omentectomy, followed by the tumor stage. Patients who underwent omentectomy were more likely to be managed in Stage I than those who underwent lymphadenectomy. After PSM-IPTW adjustment, the inclusion of omentectomy in the initial surgical procedure did not demonstrate a beneficial impact on CSS compared with those who did not undergo the procedure. Exploratory subgroup analysis indicated that the performance of omentectomy improved 5-year CSS in Stage II–IIIA patients. In the sensitive analyses for various tumor stages, omentectomy appeared to benefit only Stage II patients. However, patients across various stages seemed to benefit from the performance of lymphadenectomy, irrespective of the performance of omentectomy on them.

**Conclusion:**

Routine omentectomy may not be associated with survival benefit for patients with a grossly normal-appearing omentum, especially for those with clinical Stage I epithelial ovarian cancers.

## Introduction

Epithelial ovarian cancer (EOC) represents the most lethal female reproductive system–associated malignancy, contributing to more than 300,000 incidences and approximately 200,000 deaths worldwide in 2020 (1). EOC is a heterogeneous disease, encompassing four major histological categories: serous, mucinous, endometrioid, and clear cell (2). Comprehensive surgical staging is routinely performed in presumed early-stage EOC disease, including systemic exploration, total abdominal hysterectomy (TAH) with bilateral salpingo-oophorectomy (BSO) if no fertility-sparing is required, omentectomy, peritoneal biopsy, and/or lymphadenectomy (3). Patients with EOC higher than FIGO Stage IA are commonly administered adjuvant platinum-based chemotherapy, while postoperative prognosis depends mainly on the FIGO stage, tumor grade, and residual tumor volume. Patients who are diagnosed at Stages I or II have 5-year survival rates of 70%–90%. Regretfully, about 75% of patients are diagnosed at FIGO Stage III or IV with extensive metastasis and have a 5-year survival rate of less than 50% (4, 5). Although major improvements have been achieved in patient survival rates in many types of cancer, only a modest improvement has been accomplished in EOC even after a breakthrough therapy targeting poly ADP ribose polymerase (PARP) via inhibitors (6) or the FDA-approved bevacizumab (7), possibly due to the development of platinum resistance (8). As such, the tumor microenvironment (TME) becomes an attractive therapeutic target, which has been the focus of intensive research in recent years (9). Of particular interest is the omental tumor microenvironment (10). As a visceral adipose tissue with unique immune functions, the omentum was recognized to play an important role in response to the invasion of foreign bodies, promoting wound healing and tissue recovery (11, 12). Accordingly, gynecological oncologists and researchers started to focus their attention on whether immunological properties within the omentum could be utilized to fight the recurrence of ovarian cancer (10). If so, leaving the normal omentum behind at the time of surgery for early-stage EOC may be theoretically beneficial.

Omentectomy was historically included as part of the primary surgery of EOC because these cancer cells seemed to have a predisposition to invade the omentum (13). Certainly, the majority of ovarian cancer patients with macroscopic omental metastasis, FIGO Stage IIIA2-IVB, will succumb to their disease (14). Thus, in these patients, the omentum should be removed as part of complete cytoreduction to improve their chances of survival. However, occult omental metastases in otherwise EOC confined to ovaries have led to the consideration of omentectomy both for the purpose of concise staging and for its possible therapeutic benefit (15). With the exploration of targeted therapy and precision medicine for ovarian cancer, the role of complete removal of a grossly normal omentum becomes unclear (16). Nevertheless, there is no prospective or adequate retrospective studies to answer the question whether the removal of a macroscopically normal omentum during the surgical procedure of EOC is beneficial, neutral, or even detrimental to the patient.

As an extension of these considerations, we conducted an analysis of the Surveillance, Epidemiology, and End Results (SEER) database to determine the factors associated with the performance of omentectomy and what, if any, impact omentectomy had on cause-specific survival (CSS) in patients without a macroscopic spread beyond the pelvis.

## Methods

### Study population

We conducted a retrospective analysis for patients with epithelial ovarian cancer of predominantly or purely serous, clear cell, endometrioid, and mucinous histology. The SEER database (SEER*Stat 8.3.9.2), which contains the data of cancer patients from 17 regional registries (https://seer.cancer.gov/seerstat/), was employed for the analysis. We queried the 2021 release of the SEER database covering the 2004–2018 period, when modern staging information became available in SEER. Ovarian cancer was confirmed by the histology of a hysterectomy specimen and based on the WHO International Classification of Diseases for Oncology, third edition (ICD-O-3) morphology codes as follows: 8441-serous cystadenocarcinoma, NOS, 8460-papillary serous cystadenocarcinoma, 8461-serous surface papillary carcinoma, 8462-papillary serous cystadenocarcinoma, 8463-serous surface papillary carcinoma, 8310-clear cell carcinoma, 8330-endometrioid carcinoma, 8382-endometrioid adenocarcinoma, secretory variant, 8383-endometrioid adenocarcinoma, ciliated cell variant, 8470-mucinous cystadenocarcinoma, 8471-papillary mucinous cystadenocarcinoma, NOS, 8472-mucinous cystadenocarcinoma, 8473-seromucinous carcinoma, 8480-mucinous adenocarcinoma, 8481-mucin-producing adenocarcinoma, and 8482-mucinous adenocarcinoma, endocervical type. Cancer stage was based on the revised FIGO stage in 2014, in which IIIA was defined as tumor involving one or both ovaries or fallopian tubes, or peritoneal cancer, with a cytologically or histologically confirmed spread to the peritoneum outside the pelvis and/or metastasis to the retroperitoneal lymph nodes (17). Based on site-specific surgery codes, women who underwent at least unilateral salpingo-oophorectomy (site-specific surgery codes 25–80) were selected, and information including whether lymphadenectomy or omentectomy was performed on them was obtained. Performance of omentectomy during surgery was the focus of our study. Because all data included in the SEER database is publicly available online, this study did not require Institutional Review Board approval or informed consent by the study subjects. However, we obtained permission to access the SEER program data from the US National Cancer Institute (reference number: 22756-Nov2020).

**Figure 1:**
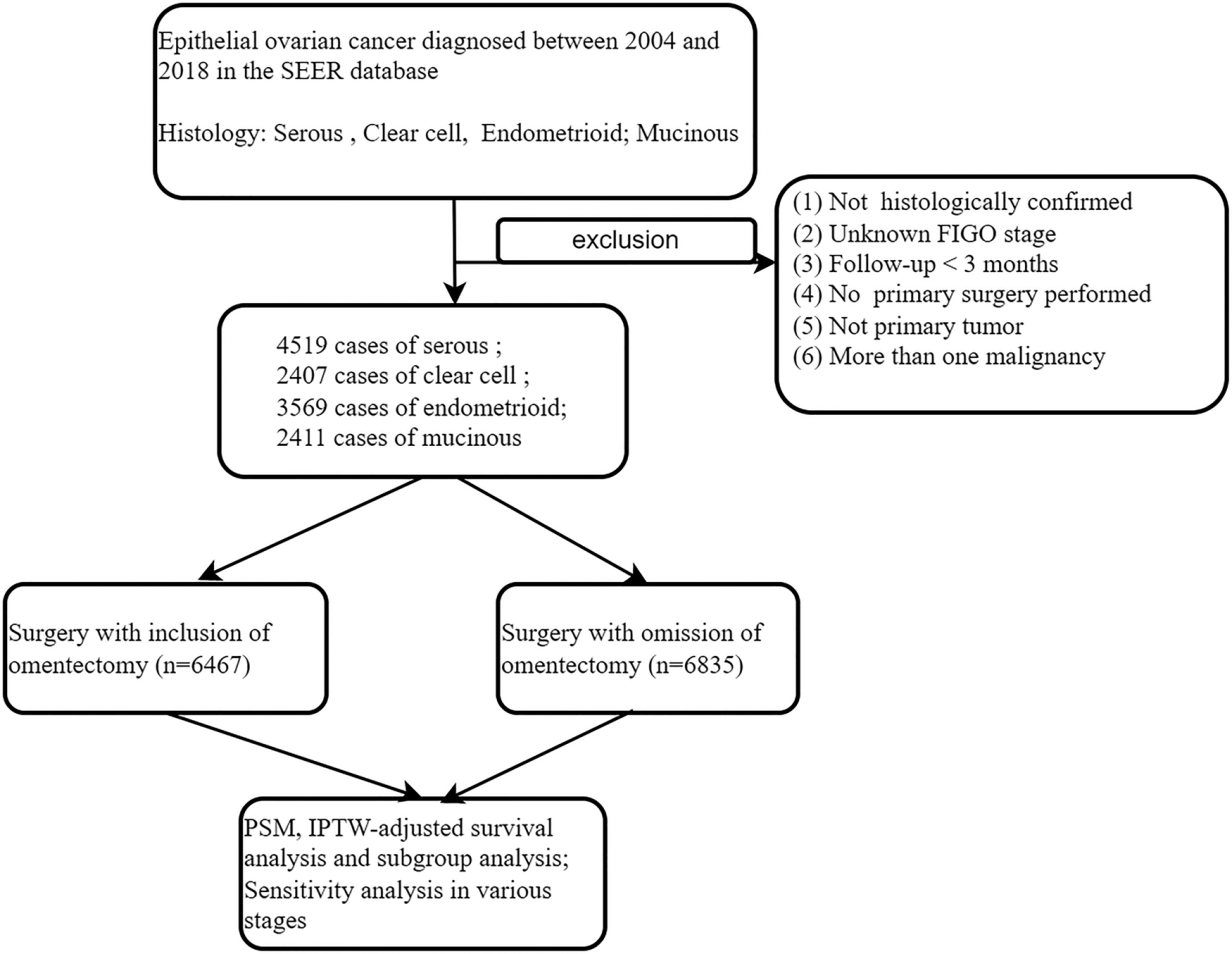
Eligibility, inclusion, and exclusion criteria of the study population.

The exclusion criteria are listed as follows: (i) patients with more than one malignancy or secondary tumor; (ii) missing information on patients' age, cancer stage, or survival period; (iii) those with the surgery code “local tumor excision or destruction; surgery NOS’’ were excluded, given the fact that we could not identify the scope of the surgical procedure performed. (iv) Stage IIIB–IVB patients were excluded because, in them, the omentum was usually involved. A landmark survival time of 3 months was applied in order to account for immortal time bias. These procedures were demonstrated as detailed in the diagram of Figure 1.

### Variable record and cohort definition

Demographic information of the patients encompassed age (<60, 60–60, and  > 70), year of diagnosis (2004–2008, 2009–2013, and 2014–2018), marital status (married, single/unmarried, divorced/separated, widowed, and unknown), race (Whites, non-Whites, and others), median household income, and serum CA125 level (elevated, normal, or not documented). Tumor characteristics included histology subtypes (endometrioid, serous, clear cell, and mucinous), stage (I–IIIA), grade (Grade I, well differentiated; Grade II, moderately differentiated; Grade III, poorly differentiated; Grade IV, undifferentiated; unknown grade), tumor size (<50, 50–100, >100 mm, unknown), and tumor laterality (unilateral, bilateral). Treatment data included hysterectomy, salpingo-oophorectomy, omentectomy, lymphadenectomy, and adjuvant chemotherapy.

### Outcome measures

Cause-specific survival (CSS) was evaluated for outcome analysis. CSS was defined as the interval from final diagnosis to death due to endometrial cancer. The primary endpoints were estimated as 3-, 5-year and 10-year CSS rates. Patients who survived at the last follow-up were censored.

### Statistical analysis

Continuous variables were described as median [interquartile range (IQR)], while categorical variables were demonstrated as frequency. Baseline characteristics were compared in terms of both pre- and post-matching with chi-square test analysis, in which the statistical significance in proportion differences with a *p* value <0.05 was considered unbalanced. Then, a binary logistic regression model was fitted, and all the pre-/intra-operative factors with a *P* < 0.05 in the univariable analysis were entered into the initial model, and a conditional backward method was used with a final stopping rule of *P* < 0.05. The Hosmer–Lemeshow test was used to assess the goodness-of-fit in the final model, and a *P* > 0.05 was interpreted as a good-fit model. These significant factors identified from binary logistic regression were utilized in subsequent analysis. In an attempt to identify the specific patterns for patient and tumor demographics for the performance of omentectomy, a recursive partitioning analysis was performed to construct a regression-tree model for risk patterns (18). Subsequently, those factors significant for performing omentectomy in the binary logistic regression analysis were entered in the final model, and the chi-square automatic interaction detector method was used for the model with a stopping rule of three layers. The determined nodes in this analysis were utilized in subsequent sensitive analysis.

To explore the performance of omentectomy on survival impact on EOC patients, multiple imputations by chained equations were conducted to control potential bias caused by confounding factors. First, we used a propensity score adjustment by inverse probability of treatment weighting (IPTW) to maximally reduce the differences between the performance and the non-performance of omentectomy. Specifically, the propensity score was calculated using a logistic regression model based on the above-analyzed characteristics. Stratified by performance or non-performance of omentectomy, the propensity score matching (PSM) method was employed through the nearest neighbor-matching with a caliper value of 0.5 for 1:1 matching. Afterward, IPTW was calculated as 1/PS (19, 20) in the omentectomy-performed group, whereas IPTW was calculated as 1/(1-PS) in the cohort with no omentectomy procedure. Stabilization of IPTW was performed by multiplying the standard IPTW by the probability of undergoing the surgery that each patient received (21). Prior to and after IPTW adjustment, univariate analysis (UVA) of the effect of patient characteristics on CSS was conducted using the Kaplan–Meier (KM) method, with the log-rank method for the evaluation of significance. Multivariable analysis (MVA) was performed by using the Cox proportional hazards regression model. Covariates included in the MVA model were selected if they were found significant in the UVA model. Next, we conducted exploratory subgroup analyses and evaluated heterogeneity as the subgroups were presumed to have been subjected to similar conditions (22). Quantification of heterogeneity was evaluated by using the *I*^2^ statistic and the Cochran Q test (23). Random-effects models were used when study heterogeneity was high (*I*^2 ^> 50%) and fixed-effects models were employed when heterogeneity was low (*I*^2 ^≤ 50%) (24). In the final sensitive analysis, Kaplan–Meier plotting was used to illustrate CSS rates based on performance of omentectomy in selected subgroups. Statistical analyses were performed by using STATA-MP (version 17.0, College Station, TX, USA), SPSS (version 22.0; SPSS, Chicago, IL, USA), and R software (version 3.6.3; http://www.r-project.org/). Two-sided hypotheses were used for statistical analysis, and a *P* < 0.05 was considered statistically significant.

## Results

### Descriptive characteristics of the study population and survival outcomes among all subgroups

According to the set criteria, the data of a total of 13,302 patients who were diagnosed with epithelia ovarian cancer as the primary malignancy and who underwent at least unilateral salpingo-oophorectomy were extracted for the 2004 and 2018 period. Table 1 depicts the demographic and clinical characteristics of these patients and survival outcomes in the whole group and subgroups. The cohort comprised 3,569 endometrioid, 4,915 serous, 2,407 clear cell, and 2,411 mucinous cancer patients, among of which the best survival outcomes were observed for those with endometrioid cancer irrespective of stage. The median age at initial diagnosis was 56 years old [interquartile range (IQR): 47–65 years old] with a median follow-up period of 67 months [interquartile range (IQR): 33–116 months]. The 3-, 5-year, and 10-year follow-up were completed in 72.65%, 54.44%, and 23.21% of all participants, respectively. Correspondingly, the 3-, 5-year, and 10-year CSS rates were 91.35%, 85.99%, and 77.87% for the whole cohort. In multivariable analysis with correction for other covariates (Table 1), increasing tumor size and patients' age, year of diagnosis between 2004 and 2008, progression of disease stage, higher tumor grade, and bigger tumor volume were related to poor survival. Patients with white race composed of the large proportion in the whole cohort and posed better CSS outcomes than those of black race. Other covariates such as median household income and hysterectomy were not evidently associated with survival outcome. Of note, the performance of lymphadenectomy including lymph node biopsy represented the majority (69.59%) of the whole cohort, providing a beneficial effect on CSS. For instance, 5-year CSS for patients in whom more than four regional lymph nodes were removed was 89.3%, which was an almost 10% improvement compared with those in whom lymphadenectomy was not performed (79.53%). Comparably, although 48.62% (6467/13,302) of patients underwent omentectomy, only 3% absolute improvement in CSS at the 3-, 5-, and 10-year follow-up period was observed, without statistical significance based on multivariate analysis. This result prompted us to further explore the survival impact of omentectomy on EOC patients.

**Table 1:** Estimated 3-, 5-, and 10-year cause-specific survival for EOC patients stratified by clinical–pathological characteristics.

		3-year CSS			5-year CSS			10-year CSS		
Characteristics	Num	Estimate, % (95%CI)	HR (95%CI)	*P*	Estimated, % (95%CI)	HR (95%CI)	*P*	Estimated, % (95%CI)	HR (95%CI)	*P*
Total	13,302	91.35% (90.83–91.84)			85.99% (85.33–86.63)			77.87% (77.01–78.71)		
Age group (years)										
<60	8,096	93.49% (92.90–94.03)	Reference		89.15% (88.38–89.86)	Reference		82.11% (81.08–83.09)	Reference	
60–70	3,273	90.76% (89.65–91.75)	1.22 (1.05–1.43)	0.012	84.88% (83.47–86.19)	1.18 (1.04–1.34)	0.009	75.63% (73.79–77.37)	1.18 (1.06–1.32)	**0** **.** **003**
>70	1,933	83.24% (81.39–84.92)	2.09 (1.76–2.48)	<0.001	74.34% (72.11–76.42)	1.99 (1.72–2.29)	<0.001	63.90% (61.27–66.40)	1.82 (1.61–2.06)	**<0** **.** **001**
Year of diagnosis										
2004–2008	3,859	90.71% (89.75–91.59)	Reference		85.20% (84.02–86.29)	Reference		77.60% (76.21–78.92)	Reference	
2009–2013	4,321	91.47% (90.59–92.27)	0.81 (0.67–0.98)	0.028	86.12% (85.03–87.13)	0.83 (0.72–0.97)	0.015	71.06% (69.33–72.71)	0.89 (0.78–1.01)	0.060
2014–2018	5,122	91.84% (90.95–92.66)	0.70 (0.57–0.86)	<0.001	86.79% (85.55–87.93)	0.71 (0.60–0.85)	<0.001	85.08% (83.69–86.35)	0.73 (0.62–0.85)	**<0** **.** **001**
Race										
Whites	10,729	91.53% (90.96–92.06)	Reference		86.10% (85.36–86.80)			77.70% (76.74–78.63)	Reference	
Others	1,853	92.29% (90.88–93.48)	0.96 (0.79–1.16)	0.654	88.00% (86.25–89.55)	0.95 (0.81–1.12)	0.537	82.07% (79.82–84.09)	0.90 (0.78–1.04)	0.155
Non-Whites	720	86.35% (83.45–88.77)	1.63 (1.30–2.04)	<0.001	79.30% (75.80–82.36)	1.55 (1.28–1.87)	<0.001	70.06% (65.84–73.86)	1.50 (1.26–1.78)	**<0** **.** **001**
Marital status										
Married	7,149	92.04% (91.35–92.67)	Reference		87.10% (86.22–87.92)			79.10% (77.94–80.21)	Reference	
Widowed	1,235	84.46% (82.19–86.46)	1.29 (1.07–1.56)	0.009	76.27% (73.56–78.74)	1.32 (1.13–1.55)	<0.001	65.42% (62.18–68.46)	1.33 (1.16–1.54)	**<0** **.** **001**
Single/unmarried	3,005	91.93% (90.84–92.90)	1.07 (0.91–1.26)	0.437	87.08% (85.69–88.35)	1.09 (0.95–1.24)	0.235	79.88% (78.08–81.55)	1.08 (0.96–1.22)	0.198
Divorced/separated	1,386	91.94% (90.29–93.32)	0.94 (0.76–1.17)	0.572	85.60% (83.43–87.50)	1.05 (0.88–1.24)	0.597	77.29% (74.51–79.81)	1.03 (0.89–1.20)	0.677
Unknown	527	93.35% (90.67–95.27)	0.75 (0.52–1.08)	0.119	88.97% (85.59–91.59)	0.78 (0.58–1.04)	0.093	81.82% (77.30–85.52)	0.80 (0.62–1.03)	0.087
Median income										
<$50,000	1,386	89.62% (87.81–91.18)	Reference		82.69% (80.40–84.74)			73.56% (70.63–76.25)	Reference	
$50,000 – $65,000	4,032	90.87% (89.90–91.75)	1.06 (0.87–1.30)	0.569	85.31% (84.08–86.44)	0.99 (0.84–1.17)	0.899	76.06% (74.42–77.62)	1.01 (0.87–1.17)	0.891
>$65,000	7,884	91.92% (91.26–92.54)	0.93 (0.76–1.13)	0.455	86.96% (86.12–87.76)	0.89 (0.76–1.04)	0.151	79.52% (78.43–.80.56)	0.95 (0.82–1.09)	0.431
Grade										
I	2,584	97.13% (96.30–97.74)	Reference		94.85% (93.82–95.72)			91.30% (89.85–92.55)	Reference	
II	2,894	93.76% (92.77–94.62)	1.87 (1.39–2.49)	<0.001	90.02% (88.76–91.14)	1.590 (1.26–2.00)	<0.001	83.29% (81.58–84.85)	1.59 (1.31–1.94)	**<0** **.** **001**
III	3,530	87.21% (85.99–88.33)	3.18 (2.40–4.21)	<0.001	79.43% (77.92–80.84)	2.69 (2.16–3.36)	<0.001	68.11% (66.26–69.89)	2.67 (2.20–3.22)	**<0** **.** **001**
IV	1,684	87.24% (85.48–88.80)	3.03 (2.24–4.11)	<0.001	78.68% (76.43–80.74)	2.49 (1.95–3.17)	<0.001	66.74% (63.92–69.39)	2.44 (1.98–3.01)	**<0** **.** **001**
Unknown	2,610	91.24% (90.02–92.31)	2.22 (1.66–2.97)	<0.001	86.46% (84.95–87.83)	1.92 (1.52–2.42)	<0.001	79.91% (78.00–81.68)	1.84 (1.50–2.24)	**<0** **.** **001**
Laterality										
Unilateral	10,979	92.22% (91.68–92.73)	Reference		87.83% (87.14–88.49)			81.15% (80.24–82.02)	Reference	
Bilateral	2,323	87.27% (85.78–88.63)	1.08 (0.92–1.26)	0.362	77.52% (75.61–79.30)	1.15 (1.01–1.31)	0.034	63.31% (60.96–65.56)	1.22 (1.09–1.36)	**<0** **.** **001**
Histology										
Endometrioid	3,569	94.99% (94.18–95.69)	Reference		91.80% (90.76–92.73)			86.47% (85.04–87.77)	Reference	
Clear cell	2,407	88.18% (86.75–89.47)	2.21 (1.76–2.74)	<0.001	83.15% (81.44–84.71)	2.00 (1.67–2.39)	<0.001	76.51% (74.41–78.47)	1.77 (1.51–2.08)	**<0** **.** **001**
Serous	4,915	89.45% (88.50–90.32)	0.96 (0.79–1.17)	0.686	81.10% (79.86–82.27)	1.16 (0.99–1.36)	0.073	69.05% (67.48–70.56)	1.29 (1.13–1.48)	**<0** **.** **001**
Mucinous	2,411	92.96% (91.80–93.95)	2.01 (1.60–2.53)	<0.001	90.21% (88.82–91.43)	1.71 (1.40–2.08)	<0.001	85.30% (83.49–86.93)	1.56 (1.31–1.85)	**<0** **.** **001**
AJCC stage										
IA	5,051	96.02% (95.41–96.55)	Reference		93.48% (92.68–94.18)			89.28% (88.19–90.27)	Reference	
IB	365	96.67% (94.06–98.14)	0.91 (0.49–1.69)	0.756	91.91% (88.26–94.46)	1.17 (0.76–1.78)	0.478	84.41% (79.36–88.31)	1.22 (0.87–1.71)	0.253
IC	3,578	92.10% (91.11–92.98)	1.97 (1.61–2.40)	<0.001	87.84% (86.61–88.97)	1.76 (1.50–2.07)	<0.001	80.40% (78.76–81.93)	1.71 (1.48–1.97)	**<0** **.** **001**
I,NOS	158	93.86% (88.53–96.76)	1.36 (0.70–2.67)	0.364	90.11% (83.58–94.14)	1.29 (0.74–2.25)	0.370	83.33% (75.13–89.03)	1.40 (0.88–2.23)	0.152
IIA	813	88.06% (85.48–90.21)	2.94 (2.24–3.86)	<0.001	81.32% (78.13–84.10)	2.48 (1.98–3.11)	<0.001	71.48% (67.54–75.04)	2.32 (1.90–2.83)	**<0** **.** **001**
IIB	1,135	86.61% (84.37–88.55)	3.15 (2.48–4.01)	<0.001	77.84% (75.07–80.34)	2.95 (2.43–3.58)	<0.001	66.28% (62.93–69.40)	2.79 (2.35–3.30)	**<0** **.** **001**
IIC	1,077	82.89% (80.43–85.06)	4.06 (3.21–5.13)	<0.001	71.70% (68.72–74.45)	3.65 (3.02–4.42)	<0.001	57.04% (53.57–60.35)	3.40 (2.89–4.01)	**<0** **.** **001**
II,NOS	138	82.95% (75.27–88.42)	3.27 (2.07–5.17)	<0.001	68.55% (59.58–75.93)	3.58 (2.53–5.08)	<0.001	57.14% (47.46–65.69)	3.20 (2.34–4.37)	**<0** **.** **001**
IIIA	987	80.69% (77.83–83.22)	4.71 (3.69–6.01)	<0.001	69.53% (66.13–72.67)	4.17 (3.41–5.10)	<0.001	56.42% (52.64–60.02)	4.08 (3.42–4.86)	**<0** **.** **001**
Tumor size (mm)										
<50	2,349	93.91% (92.80–94.85)	Reference		89.15% (87.68–90.46)			81.70% (79.71–83.52)	Reference	
50–100	3,109	91.48% (90.38–92.46)	1.19 (0.96–1.48)	0.110	86.38% (84.99–87.65)	1.06 (0.89–1.26)	0.514	78.02% (76.20–79.72)	1.03 (0.89–1.20)	0.696
>100	5,960	91.31% (90.52–92.03)	1.28 (1.05–1.57)	0.014	86.04% (85.04–86.99)	1.21 (1.03–1.42)	0.019	78.42% (77.11–79.66)	1.15 (1.00–1.32)	**0** **.** **044**
Unknown	1,884	88.25% (86.66–89.66)	1.64 (1.31–2.05)	<0.001	81.61% (79.67–83.38)	1.48 (1.24–1.77)	<0.001	71.99% (69.61–74.22)	1.38 (1.19–1.61)	**<0** **.** **001**
Omentectomy										
Yes	6,467	92.42% (91.71–93.07)	Reference		87.33% (86.41–88.19)			79.58% (78.37–80.73)	Reference	
No	6,835	90.34% (89.57–91.05)	1.10 (0.15–7.84)	0.928	84.72% (83.76–85.63)	0.71 (0.10–5.09)	0.736	76.24% (75.00–77.43)	1.20 (0.30–4.85)	0.794
Salpingo-oophorectomy										
USO	1,152	91.69% (89.85–93.21)	Reference		87.86% (85.63–89.77)			82.57% (79.75–85.03)	Reference	
BSO	5,675	90.07% (89.21–90.86)	0.82 (0.65–1.05)	0.109	84.07% (82.99–85.09)	0.87 (0.71–1.06)	0.167	74.96% (73.56–76.29)	0.95 (0.79–1.15)	0.599
Unknown	6,475	92.41% (91.70–93.06)	0.85 (0.12–6.13)	0.869	87.33% (86.41–88.19)	0.59 (0.08–4.26)	0.603	79.56% (78.35–80.71)	1.08 (0.27–4.40)	0.911
Lymphadenectomy										
1–3 regional	1,210	91.33% (89.49–92.86)	Reference		85.48% (83.15–87.51)			76.71% (73.70–79.42)	Reference	
≥4 regional	7,962	93.80% (93.22–94.33)	0.82 (0.65–1.02)	0.076	89.30% (88.53–90.02)	0.81 (0.68–0.98)	0.025	81.80% (80.75–82.80)	0.86 (0.71–0.96)	**0** **.** **015**
SLN biopsy/removed	84	94.48% (85.96–97.89)	0.76 (0.28–2.06)	0.582	89.63% (79.47–94.92)	0.83 (0.39–1.78)	0.637	77.57% (63.90–86.59)	1.13 (0.63–2.02)	0.687
None/unknown	4,046	86.42% (85.27–87.50)	1.70 (1.36–2.12)	<0.001	79.53% (78.13–80.86)	1.55 (1.29–1.85)	<0.001	70.77% (69.07–72.40)	1.43 (1.22–1.67)	**<0** **.** **001**
Chemotherapy										
Yes	8,101	89.71% (88.99–90.39)	Reference		82.81% (81.88–83.69)			73.15% (71.97–74.29)	Reference	
No/Unknown	5,201	93.90% (93.17–94.54)	0.89 (0.77–1.04)	0.134	90.93% (90.04–91.75)	0.77 (0.68–0.873)	<0.001	85.23% (84.03–86.35)	0.78 (0.70–0.88)	**<0** **.** **001**
CA-125 level										
Positive/elevated	4,919	90.05% (89.11–90.92)	Reference		84.10% (82.88–85.24)			75.24% (73.60–76.79)	Reference	
Negative/normal	1,724	94.65% (93.36–95.69)	0.64 (0.50–0.82)	<0.001	90.57% (88.82–92.05)	0.721 (0.59–0.88)	0.001	84.48% (82.00–86.65)	0.76 (0.63–0.91)	**0** **.** **003**
Not documented	6,659	91.45% (90.74–92.12)	0.78 (0.66–0.94)	0.007	86.19% (85.30–87.03)	0.81 (0.70–0.94)	0.005	78.09% (76.97–79.17)	0.85 (0.75–0.97)	**0** **.** **013**

EOC, epithelial ovarian cancer; HR, hazard ratio based on multivariate analysis; SLN, sentinel lymph node; USO, unilateral salpingo-oophorectomy; BSO, bilateral salpingo-oophorectomy; CA125, carbohydrate antigen 125; CSS, cause-specific survival.

### Exploration of the performance of omentectomy among subgroups and cause-specific survival analysis after PSM–IPTW adjustment by the non-performance of omentectomy

To further explore the association of omentectomy among various clinicopathologic parameters, we stratified the cohort by way of performance or non-performance of omentectomy, as illustrated in Table 2. Before PSM and IPTW adjustment, most baseline characteristics were found to be significantly unbalanced based on univariate analysis. Patients who underwent omentectomy tended to be younger than 60 years of age, were diagnosed between 2004 and 2008, were non-Whites, excluding others, had a higher household income, had tumor involving both ovaries and bigger than 50 mm in diameter, had a histology of clear cell, endometrioid and mucinous subtypes, belonged to the group of Stage I, and who underwent both lymphadenectomy and hysterectomy. In binary logistic analysis, the performance of lymphadenectomy, early-stage tumor, and a bigger tumor size was linked to an increased likelihood of omentectomy (all *P* < 0.05). In the regression-tree model with recursive partitioning analysis, Hosmer–Lemeshow test indicated a good-fit model (*P* = 0.331). The procedure of lymphadenectomy was found to be the strongest factor to distinguish the performance of omentectomy, followed by the tumor stage (Supplementary Figure S1). Notably, in the omentectomy group, lymphadenectomy patients in whom more than four regional lymph nodes were removed constituted nearly 70%. After PSM and IPTW adjustment by omentectomy, all baseline characteristics were well balanced with a *P* value > 0.05. The results are demonstrated in Table 2.

**Table 2:** Baseline characteristics before and after IPTW adjusted by omentectomy.

Characteristics	Omentectomy, *n* % (preadjusted)			Binary logistic analysis		Omentectomy, % (IPTW adjusted)		
	No	Yes	*P*-value	OR (95%CI)	*P*-value	No (%)	Yes (%)	*P*-value
Age group (years)								
60–70	1,694 (24.78)	1,579 (24.42)	0.000**			24.78	24.66	0.455
<60	4,051 (59.27)	4,045 (62.55)				60.11	60.97	
>70	1,090 (15.95)	843 (13.04)				15.11	14.38	
Year of diagnosis					**<0** **.** **001**			
2004–2008	1,891 (27.67)	1,968 (30.43)	0.002**	1		29.14	29.18	1.000
2009–2013	2,262 (33.09)	2,059 (31.84)		1.21 (1.10–1.32)	<0.001	32.44	32.36	
2014–2018	2,682 (39.24)	2,440 (37.73)		0.99 (0.91–1.07)	0.729	38.42	38.46	
Race								
Non-Whites	425 (6.22)	295 (4.56)	0.000**			5.86	4.98	0.056
Others^a^	911 (13.33)	942 (14.57)				13.53	14.15	
Whites	5,499 (80.45)	5,230 (80.87)				80.61	80.87	
Marital status								
Divorced/separated	712 (10.42)	674 (10.42)	0.004**			10.42	10.42	0.387
Married	3,583 (52.42)	3,566 (55.14)				53.24	54.61	
Single/unmarried	1,570 (22.97)	1,435 (22.19)				22.77	22.12	
Unknown	294 (4.30)	233 (3.60)				4.12	3.59	
Widowed	676 (9.89)	559 (8.64)				9.45	9.25	
Median househ = old income								
$50,000 – $65,000	2,070 (30.29)	1,962 (30.34)	0.000**			29.84	30.96	0.061
<$50,000	790 (11.56)	596 (9.22)				11.12	9.96	
>$65,000	3,975 (58.16)	3,909 (60.45)				59.04	59.07	
Grade								
I	1,309 (19.15)	1,275 (19.72)	0.007**			18.89	19.46	0.178
II	1,438 (21.04)	1,456 (22.51)				21.61	22.10	
III	1,849 (27.05)	1,681 (25.99)				27.34	26.38	
IV	833 (12.19)	851 (13.16)				12.23	13.18	
Unknown	1,406 (20.57)	1,204 (18.62)				19.92	18.88	
Tumor laterality								
Bilateral ovaries	1,279 (18.71)	1,044 (16.14)	0.000**			18.04	17.99	0.946
Unilateral ovary	5,556 (81.29)	5,423 (83.86)				81.96	82.01	
Histology								
Clear cell	1,134 (16.59)	1,273 (19.68)	0.000**			17.70	18.00	0.202
Endometrioid	1,795 (26.26)	1,774 (27.43)				27.16	26.48	
Mucinous	1,215 (17.78)	1,196 (18.49)				17.27	18.56	
Serous	2,691 (39.37)	2,224 (34.39)				37.87	36.96	
AJCC Stage					**<0** **.** **001**			
I,NOS	86 (1.26)	72 (1.11)	0.000**	1		1.16	1.12	0.996
IA	2,540 (37.16)	2,511 (38.83)		1.30 (0.91–1.86)	0.149	37.40	36.88	
IB	180 (2.63)	185 (2.86)		1.35 (1.16–1.57)	<0.001	2.79	2.80	
IC	1,696 (24.81)	1,882 (29.10)		1.35 (1.05–1.73)	0.019	26.90	26.75	
II,NOS	86 (1.26)	52 (0.80)		1.44 (1.24–1.68)	<0.001	1.04	1.02	
IIA	426 (6.23)	387 (5.98)		0.93 (0.64–1.36)	0.715	6.17	6.15	
IIB	659 (9.64)	476 (7.36)		1.24 (1.02–1.50)	0.030	8.60	8.80	
IIC	594 (8.69)	483 (7.47)		0.93 (0.78–1.11)	0.414	8.25	8.42	
IIIA	568 (8.31)	419 (6.48)		1.06 (0.89–1.27)	0.513	7.69	8.05	
Tumor Size (mm)					**0** **.** **001**			
50–100	1,551 (22.69)	1,558 (24.09)	0.000**	1		23.40	23.46	0.982
<50	1,351 (19.77)	998 (15.43)		0.92 (0.81–1.04)	0.182	17.43	17.17	
>100	2,874 (42.05)	3,086 (47.72)		1.21 (1.08–1.35)	0.001	45.03	45.21	
Unknown	1,059 (15.49)	825 (12.76)		1.19 (1.05–1.34)	0.005	14.14	14.16	
Total hysterectomy					0.001			
No/unknown	1,665 (24.36)	1,035 (16.00)	0.000**	1		20.07	19.61	0.529
Yes	5,170 (75.64)	5,432 (84.00)		0.66 (0.61–0.73)		79.93	80.39	
Lymphadenectomy					<0.001			
1–3 regional	579 (8.47)	631 (9.76)	0.000**	1		9.09	9.11	0.995
None/unknown	2,722 (39.82)	1,324 (20.47)		1.47 (0.95–2.28)	0.082	30.33	30.14	
SLN biopsy/removed	46 (0.67)	38 (0.59)		1.31 (0.84–2.05)	0.239	0.64	0.66	
≥4 regional	3,488 (51.03)	4,474 (69.18)		0.61 (0.39–0.95)	0.028	59.95	60.09	
Chemotherapy								
No/Unknown	2,759 (40.37)	2,442 (37.76)	0.002**			39.22	37.56	0.052
Yes	4,076 (59.63)	4,025 (62.24)				60.78	62.44	
CA-125 pretreatment level								
Negative/normal	885 (12.95)	839 (12.97)	0.975			12.56	12.71	0.449
Not documented	3,428 (50.15)	3,231 (49.96)				50.43	49.35	
Positive/elevated	2,522 (36.90)	2,397 (37.07)				37.01	37.93	

SLN, sentinel lymph node; Race Others^a^: American Indian, Asian/Pacific Islander; IPTW, inverse probability treatment weighting; CA125, Carbohydrate antigen 125.

Chi-square test for univariable analysis. A binary logistic regression model for multivariable analysis. All preoperative and operative covariates with *P* < 0.05 in univariable analysis were entered in the initial model and the conditional backward method with the stopping rule of *P* < 0.05.

**means significant statistical difference.

After PSM and IPTW adjustment, univariate analysis (UVA) revealed that omentectomy patients showed improved 3- and 5-year CSS outcomes; however, multivariate analysis (MVA) also revealed similar 3-year CSS (HR 1.08, 95% CI 0.95–1.25, *P *= 0.26) and 5-year CSS (HR1.05, 95% CI 0.94–1.17, *P *= 0.406) outcomes. Prognostic factors associated with CSS in patients adjusted by omentectomy persisted, similar to all other significant factors pre-adjustment. A similar prognosis was observed between endometrioid and serous histology patients, although they showed better survival rates than clear cell and mucinous subtypes. Hysterectomy was not statistically associated with prognosis. The performance of lymphadenectomy still provided a beneficial effect on CSS, with the benefit being prominent in those in whom more than four regional lymph nodes were removed. Adjusted UVA and MVA are displayed in Table 3.

**Table 3:** Survival analysis of predicting CSS after IPTW adjusted by omentectomy in EOC patients.

Characteristics	3-year cause-specific survival				5-year cause-specific survival			
	Univariate analysis	Multivariate analysis	Univariate analysis	Multivariate analysis
Omentectomy								
Yes	Reference				Reference			
No	1.29 (1.14–1.46)	**<0** **.** **001**	1.08 (0.95–1.24)	0.260	1.23 (1.11–1.36)	**<0** **.** **001**	1.05 (0.94–1.17)	0.406
Age group (years)								
<60	Reference				Reference			
60–70	1.43 (1.23–1.66)	**<0** **.** **001**	1.22 (1.04–1.42)	**0** **.** **016**	1.43 (1.27–1.62)	**<0** **.** **001**	1.18 (1.04–1.33)	**0** **.** **012**
>70	2.72 (2.35–3.15)	**<0** **.** **001**	2.07 (1.75–2.46)	**<0** **.** **001**	2.62 (2.32–2.95)	**<0** **.** **001**	1.98 (1.72–2.28)	**<0** **.** **001**
Year of diagnosis								
2004–2008	Reference				Reference			
2009–2013	0.92 (0.79–1.06)	0.258	0.81 (0.67–0.98)	**0** **.** **029**	0.94 (0.84–1.05)	0.282	0.83 (0.71–0.96)	**0** **.** **015**
2014–2018	0.848 (0.729–0.987)	**0** **.** **033**	0.70 (0.57–0.86)	**0** **.** **001**	0.86 (0.76–0.98)	**0** **.** **022**	0.71 (0.60–0.85)	**<0** **.** **001**
Race								
Whites	Reference				Reference			
Others	0.91 (0.76–1.10)	0.329	0.95 (0.79–1.16)	0.661	0.87 (0.74–1.02)	0.078	0.95 (0.81–1.12)	0.540
Non-Whites	1.66 (1.34–2.07)	**<0** **.** **001**	1.63 (1.30–2.04)	**<0** **.** **001**	1.57 (1.30–1.89)	**<0** **.** **001**	1.55 (1.28–1.88)	**<0** **.** **001**
Marital status								
Married	Reference				Reference			
Widowed	2.06 (1.73–2.44)	**<0** **.** **001**	1.29 (1.07–1.56)	**0** **.** **008**	2.00 (1.74–2.31)	**<0** **.** **001**	1.32 (1.13–1.55)	**0** **.** **001**
Single/unmarried	1.02 (0.87–1.19)	0.844	1.08 (0.91–1.27)	0.395	0.99 (0.88–1.14)	0.983	1.09 (0.95–1.25)	0.205
Divorced/separated	1.01 (0.82–1.25)	0.923	0.94 (0.76–1.17)	0.591	1.11 (0.94–1.32)	0.207	1.05 (0.89–1.24)	0.583
Unknown	0.85 (0.59–1.22)	0.368	0.75 (0.52–1.08)	0.121	0.86 (0.64–1.16)	0.322	0.78 (0.58–1.04)	0.093
Median household income								
<$50,000	Reference				Reference			
$50,000–$65,000	0.89 (0.73–1.09)	0.263	1.06 (0.87–1.30)	0.560	0.85 (0.72–0.99)	**0** **.** **043**	0.99 (0.84–1.17)	0.893
>$65,000	0.78 (0.64–0.94)	**0** **.** **009**	0.93 (0.76–1.12)	0.443	0.76 (0.65–0.88)	**<0** **.** **001**	0.89 (0.76–1.04)	0.141
Rural–urban								
Urban	Reference				Reference			
Rural	1.08 (0.89–1.31)	0.457			1.14 (0.96–1.31)	0.141		
Grade								
I	Reference				Reference			
II	2.21 (1.66–2.94)	**<0** **.** **001**	1.86 (1.39–2.48)	**<0** **.** **001**	1.97 (1.57–2.47)	**<0** **.** **001**	1.59 (1.26–1.99)	**<0** **.** **001**
III	4.72 (3.64–6.13)	**<0** **.** **001**	3.16 (2.39–4.19)	**<0** **.** **001**	4.47 (3.64–5.48)	**<0** **.** **001**	2.69 (2.15–3.35)	**<0** **.** **001**
IV	4.56 (3.45–6.02)	**<0** **.** **001**	3.02 (2.23–4.09)	**<0** **.** **001**	4.31 (3.46–5.37)	**<0** **.** **001**	2.48 (1.95–3.16)	**<0** **.** **001**
Unknown	3.15 (2.38–4.15)	**<0** **.** **001**	2.22 (1.66–2.97)	**<0** **.** **001**	2.78 (2.23–3.47)	**<0** **.** **001**	1.92 (1.52–2.42)	**<0** **.** **001**
Laterality								
Unilateral	Reference				Reference			
Bilateral	1.67 (1.45–1.92)	**<0** **.** **001**	1.07 (0.91–1.25)	0.428	1.95 (1.74–2.17)	**<0** **.** **001**	1.14 (1.01–1.29)	**0** **.** **042**
Histology								
Endometrioid	Reference				Reference			
Clear cell	2.45 (2.02–2.99)	**<0** **.** **001**	2.20 (1.78–2.73)	**<0** **.** **001**	2.21 (1.87–2.61)	**<0** **.** **001**	1.99 (1.67–2.39)	**<0** **.** **001**
Serous	2.15 (1.80–2.58)	**<0** **.** **001**	0.96 (0.79–1.17)	0.701	2.45 (2.12–2.83)	**<0** **.** **001**	1.16 (0.99–1.38)	0.071
Mucinous	1.44 (1.15–1.79)	**0** **.** **001**	2.04 (1.62–2.57)	**<0** **.** **001**	1.22 (1.01–1.47)	**0** **.** **039**	1.72 (1.42–2.09)	**<0** **.** **001**
AJCC stage								
IA	Reference				Reference			
IB	0.84 (0.46–1.54)	0.562	0.89 (0.48–1.66)	0.722	1.25 (0.83–1.89)	0.280	1.16 (0.76–1.76)	0.504
IC	2.00 (1.66–2.42)	**<0** **.** **001**	1.96 (1.61–2.38)	**<0** **.** **001**	1.94 (1.66–2.27)	**<0** **.** **001**	1.76 (1.49–2.07)	**<0** **.** **001**
I,NOS	1.56 (0.80–3.04)	0.196	1.36 (0.70–2.66)	0.366	1.50 (0.86–2.62)	0.152	1.29 (0.74–2.24)	0.378
IIA	3.11 (2.41–4.02)	**<0** **.** **001**	2.90 (2.21–3.80)	**<0** **.** **001**	3.06 (2.47–3.78)	**<0** **.** **001**	2.46 (1.96–3.08)	**<0** **.** **001**
IIB	3.55 (2.85–4.44)	**<0** **.** **001**	3.12 (2.45–3.96)	**<0** **.** **001**	3.79 (3.17–4.54)	**<0** **.** **001**	2.93 (2.41–3.55)	**<0** **.** **001**
IIC	4.54 (3.69–5.59)	**<0** **.** **001**	4.02 (3.18–5.07)	**<0** **.** **001**	4.80 (4.06–5.68)	**<0** **.** **001**	3.62 (2.99–4.39)	**<0** **.** **001**
II,NOS	4.65 (2.99–7.24)	**<0** **.** **001**	3.24 (2.05–5.12)	**<0** **.** **001**	5.63 (4.02–7.87)	**<0** **.** **001**	3.56 (2.51–5.05)	**<0** **.** **001**
IIIA	5.25 (4.25–6.50)	**<0** **.** **001**	4.67 (3.66–5.96)	**<0** **.** **001**	5.71 (4.79–6.81)	**<0** **.** **001**	4.15 (3.39–5.07)	**<0** **.** **001**
Tumor size (mm)								
<50	Reference				Reference			
50–100	1.41 (1.14–1.74)	**0** **.** **002**	1.19 (0.96–1.48)	0.117	1.27 (1.07–1.51)	**0** **.** **006**	1.06 (0.89–1.26)	0.539
>100	1.44 (1.19–1.75)	**<0** **.** **001**	1.28 (1.05–1.56)	**0** **.** **017**	1.31 (1.12–1.53)	**0** **.** **001**	1.20 (1.03–1.41)	**0** **.** **023**
Unknown	1.99 (1.60–2.49)	**<0** **.** **001**	1.64 (1.31–2.05)	**<0** **.** **001**	1.78 (1.49–2.12)	**<0** **.** **001**	1.48 (1.24–1.77)	**<0** **.** **001**
Total hysterectomy								
Yes	Reference				Reference			
No/unknown	0.95 (0.81–1.11)	0.495			0.92 (0.81–1.04)	0.175		
Lymphadenectomy								
1–3 regional	Reference				Reference			
≥4 regional	0.70 (0.56–0.88)	**0** **.** **002**	0.81 (0.65–1.02)	0.070	0.71 (0.59–0.85)	**<0** **.** **001**	0.81 (0.68–0.97)	**0** **.** **024**
SLN biopsy/removed	0.63 (0.23–1.71)	0.364	0.76 (0.28–2.07)	0.585	0.71 (0.33–1.52)	0.382	0.83 (0.39–1.79)	0.644
None/unknown	1.61 (1.30–2.01)	**<0** **.** **001**	1.71 (1.37–2.14)	**<0** **.** **001**	1.47 (1.23–1.76)	**<0** **.** **001**	1.60 (1.30–1.87)	**<0** **.** **001**
Chemotherapy								
Yes	Reference				Reference			
No/Unknown	0.59 (0.51–0.67)	**<0** **.** **001**	0.90 (0.77–1.04)	0.154	0.51 (0.45–0.57)	**<0** **.** **001**	0.77 (0.68–0.88)	**<0** **.** **001**
CA-125 level								
Positive/elevated	Reference				Reference			
Negative/normal	0.53 (0.41–0.67)	**<0** **.** **001**	0.64 (0.50–0.82)	**<0** **.** **001**	0.57 (0.47–0.69)	**<0** **.** **001**	0.72 (0.59–0.88)	**0** **.** **001**
Not documented	0.87 (0.76–0.98)	**0** **.** **026**	0.79 (0.66–0.94)	**0** **.** **009**	0.86 (0.77–0.95)	**0** **.** **005**	0.81 (0.70–0.94)	**0** **.** **005**

EOC, epithelial ovarian cancer; HR, hazard ratio based on multivariate analysis; SLN, sentinel lymph node; CA125, carbohydrate antigen 125; CSS, cause-specific survival.

### Exploratory subgroup and sensitivity analyses in EOC patients stratified by the performance of omentectomy

**Figure 2:**
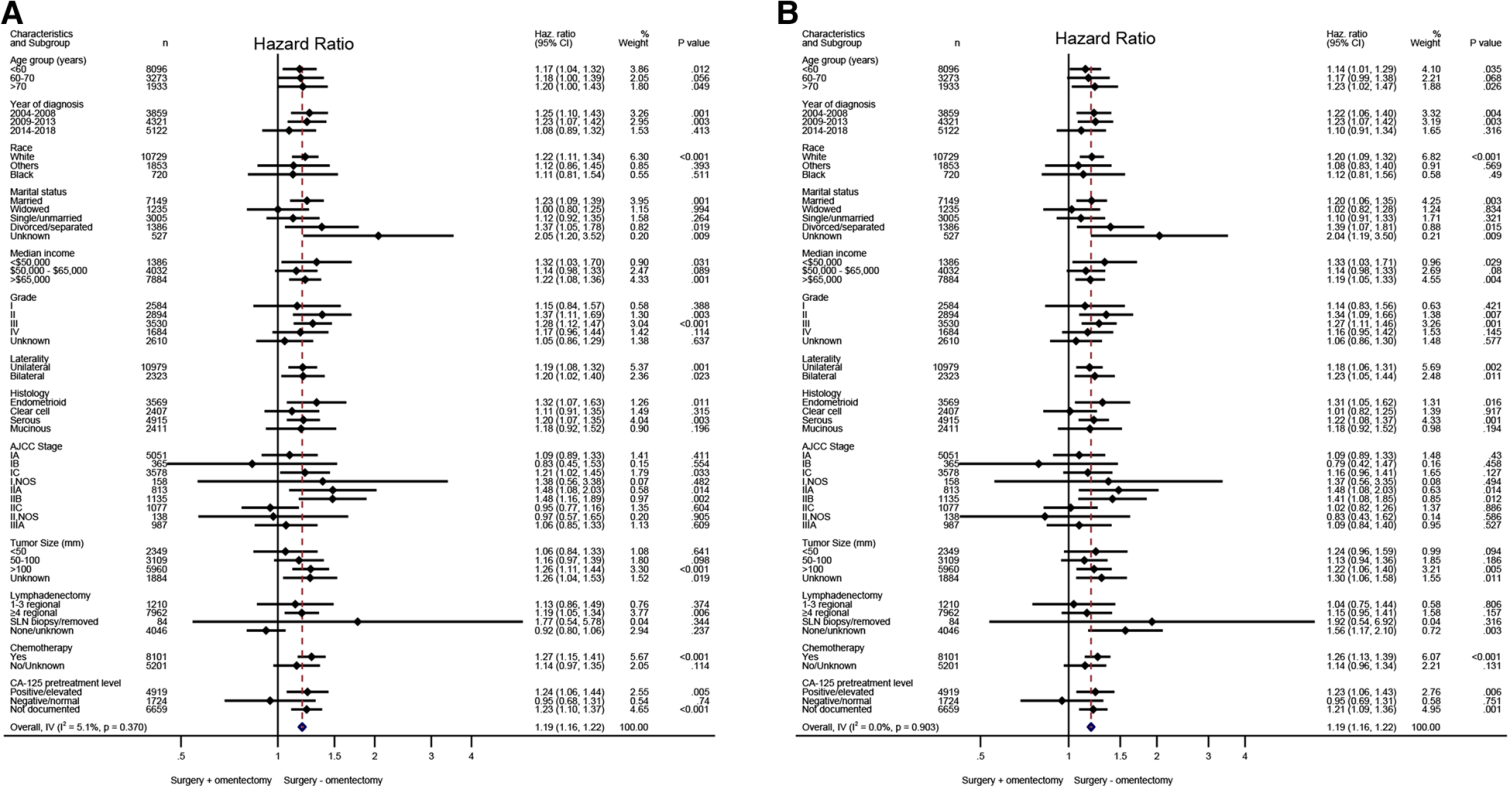
Exploratory subgroup analysis concerning omentectomy impact on survival outcome in the whole cohort. (**A**) Cause-specific survival before IPTW adjustment. (**B**) Cause-specific survival after IPTW adjustment. (**D**) Overall survival after IPTW adjustment. CI, confidence interval; HR, hazard ratio; IPTW, inverse probability of treatment weighting. The vertical solid line refers to a hazard ratio of 1.0. HR < 1 favors surgery without omentectomy and HR > 1 favors surgery with omentectomy. A score value of *P* < 0.05 indicates statistical significance.

Based on the aforementioned multivariate analysis, the performance of omentectomy was not associated with beneficial survival impact, even though survival difference was evident in univariate analysis. This inconsistent result prompted us to further explore who would possibly benefit from the procedure of omentectomy. An exploratory subgroup analysis related to prognosis was conducted in selected subgroups, as shown in the forest plot (Figure 2). Before and after matching, heterogeneity was found to be low (*I*^2 ^< 10%) in the fixed-effects model; therefore, we employed the fixed-effects model to illustrate the result. Prior to matching, survival benefit was observed from the performance of omentectomy in those patients who were younger than 60 years old, diagnosed between 2004 and 2013, those with Stage IC–IIB, tumor grade II, a histology of endometrioid, and serous subtypes, and those in whom more than four regional lymph nodes were removed (Figure 2A). After IPTW adjustment, the above prognostic factors persisted; however, patients who did undergo lymphadenectomy benefited from the performance of omentectomy (Figure 2B), underlining the impact factor in terms of lymphadenectomy and omentectomy on EOC patients.

**Figure 3:**
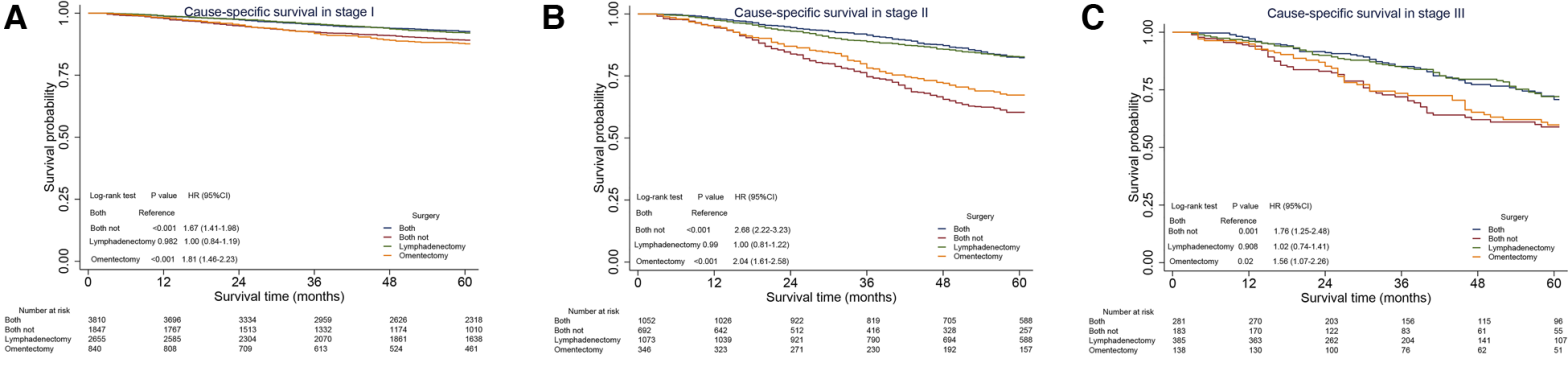
Sensitivity analysis for omentectomy and lymphadenectomy on cause-specific survival (CSS) in Stage I–IIIA epithelial ovarian cancer. (**A**) CSS in Stage I EOC. (**B**) CSS in Stage II EOC. (**C**) CSS in Stage IIIA1 EOC. CI, confidence interval; HR, hazard ratio. *P* < 0.05 indicates statistical significance.

As analyzed above, the performance of omentectomy was unbalanced in various stages vis-à-vis lymphadenectomy, with the two factors related to prognosis. Thus, we further performed sensitivity analysis to explore the impact of omentectomy and lymphadenectomy on patients’ survival stratified by disease stages. The patients were classified as follows: omentectomy alone, lymphadenectomy alone, both, and none. The performance of omentectomy showed a similar impact on CSS compared with those who did not undergo the procedure, irrespective of the performance of lymphadenectomy (Figure 3). In detail, lymphadenectomy showed a beneficial effect on those who underwent the procedure compared with those who did not undergo it. Stage I EOC patients benefited from lymphadenectomy alone compared with omentectomy alone; however, those with a combination of both did not show better survival than those with lymphadenectomy alone (Figure 3A). Significant CSS improvement after the performance of omentectomy alone was observed only in Stage II patients (Figure 3B). For Stage IIIA patients, lymphadenectomy with or without omentectomy promoted better survival rates than omentectomy alone or none of these, and no survival difference existed between the latter two patient groups (Figure 3C).

## Discussion

The current SEER database exploration was a retrospective population-based analysis with, to our knowledge, the largest sample size to evaluate factors associated with the performance of omentectomy and its impact on CSS in patients with epithelia ovarian cancer. By way of literature review, there has never been a randomized control trial explaining whether resection of a grossly normal-appearing omentum made any difference in EOC patient outcomes. An analysis of the earlier SEER database included 5,454 EOC patients who underwent omentectomy during surgery compared with 2,404 patients who did not undergo the procedure. No statistical difference was found in terms of disease-specific survival in that cohort (25). In this study, we included a larger number of cases with a relatively long follow-up period to further explore the impact of omentectomy on the survival of EOC patients.

Omentectomy was included at the time of surgery in approximately half of EOC patients. Although a marginal increase in the estimated survival rate was observed in these omentectomy EOC patients, after controlling for confounders, it was found that the performance of omentectomy was not associated with better survival. Afterward, we utilized the PMS–IPTW method to control for confounding factors and conditional landmark analysis, reducing the possibility that this conclusion suffered from selection bias and immortality bias, respectively. In subgroup and sensitive analyses, omentectomy showed a significant survival benefit for patients in Stage II; however, there was no survival difference in those with Stage I disease.

Omentectomy was initially included as a routine component of ovarian cancer surgery in the early 1960s. At that time, it was indicated that ovarian cancer patients who had undergone extensive tumor removal during operation, fared better (26). Until now, the current clinical guidelines also included omentectomy in the standard surgical procedure for all epithelial ovarian cancer patients, even in early-stage disease (3, 17, 27). For instance, the most recent NCCN guidelines recommended that for patients with disease apparently confined to the ovaries or to the pelvis (presumed Stage I/II), omentectomy should be performed to exclude higher-stage disease. For patients with disease spreading to the upper abdomen (Stage III/IV), it was recommended that omentum be removed (3). However, neither of the abovementioned clinical guidelines spoke about the specific survival impact of omentectomy on EOC patients. Omentectomy was included in most EOC cases based on the theory that omentum may harbor micrometastases and/or may be a site of recurrent disease in the future. As such, comprehensive staging mainly contributed to the determination in terms of adjuvant chemotherapy, which was based only on the results of staging surgery. However, the percentage of microscopic metastasis in a grossly normal omentum remains uncertain. Limited studies reported that the rate of occult omental metastases in EOC confined to ovaries (Stage I) ranged from 2% to 4% and then reached a higher stage to Stage III A in 3% to 11% of patients (28). However, performing an omentectomy did not result in a prolonged survival period in this condition, according to the recent French guidelines for oncology (28). In one retrospective review of 256 ovarian cancer patients with a macroscopically normal omentum, it was observed that after routine staging omentectomy, only 7 (2.7%) patients were upstaged and only one patient received adjuvant chemotherapy based on microscopic metastasis (29). Another study that enrolled 211 patients showed that only 2% of patients were upstaged and no patients were determined to be administered with chemotherapy based only on microscopic involvement of the omentum (30). That study included patients with endometrioid (37%), serous (25%), mucinous (16%), and clear cell (14%) cancer subtypes. Our exploratory subgroup analysis found that patients with serous and endometrioid histologies may benefit from the performance of omentectomy; however, no impact was found on those with mucinous and clear cell subtypes. A literature search revealed only one research showing 1 of 35 “initial” Stages IA and 33 IC ovarian mucinous cancer patients having microscopic omentum metastasis at the final histological analysis (31). Nevertheless, in those patients in whom adjuvant chemotherapy was determined, it was uncertain whether the removal of the normal omentum resulted in any potential therapeutic effect (32–34). In addition, the pathology literature suggested that for the sole purpose of staging and detecting microscopic disease, omental biopsies may probably suffice in a grossly normal-appearing omentum (34). One group of researchers suggested that, with regard to pathologic evaluation, 10 blocks could obtain a sensitivity of as high as 95% (35). Another early study reported that, for patients with endometrial and ovarian carcinomas without a macroscopic intra-abdominal lesion, just three to five omentum samples seemed sufficient for staging (36). Based on these findings, most oncologists reasoned that a careful macroscopic examination might be the most important procedure in identifying small omental metastasis.

Given that the performance of lymphadenectomy was identified as the most prominent confounding factor for omentectomy in the present analysis, we paid particular attention to the impact of omentectomy on CSS in the subgroup of lymphadenectomy and non-lymphadenectomy patients. More importantly, we further explored the survival difference among patients who underwent lymphadenectomy, omentectomy, both, or none, with the purpose of reducing the possibility of omentectomy impact on survival confounded by lymphadenectomy. The role of lymphadenectomy in ovarian cancer has been in the realm of contradiction for a long period. Many retrospective analyses, including involving large samples of patients, have indicated a survival benefit for lymphadenectomy, and accordingly, patients have been exposed to this procedure over several decades. Lymph-node metastases detected by systematic lymphadenectomy have been reported in the range of 44 to 53% in patients across all FIGO stages (37). A higher stage is associated with an increasing frequency of lymph node metastases, approximately 3%–14% being reported in early-stage EOC patients (38, 39). The core issue here is whether removal of lymph nodes should be performed only to stage the disease or whether the removal itself improves survival (40). For EOC patients in the presumed early stage, a randomized study showed that systematic lymphadenectomy facilitated better and easier detection of metastatic nodes compared with lymph node sampling, but this was not associated with improved survival (41). Another meta-analysis reported that systematic lymphadenectomy improved OS in patients with early-stage disease, even though it did not improve progression-free survival (42). In our study, lymphadenectomy was performed in a majority of Stage I–II patients, demonstrating a significant improvement compared with those without lymph node removal. The addition of omentectomy to lymphadenectomy did not improve survival rates compared with lymphadenectomy alone. In Stage II patients in whom lymphadenectomy was performed, omentectomy showed a survival benefit. Interestingly, the performance of omentectomy persistently showed no survival improvement in Stage IIIA patients, which was partly upstaged by lymphadenectomy when tumors confined to the ovaries. Chemotherapy is usually administered in Stage IIIA EOC patients, which makes the influence of omentectomy on such patients more uncertain.

Although we included the largest sample of patients in whom omentectomy was performed during surgery to investigate the impact of omentectomy on the CSS of patients with clinical Stage I–IIIA EOC, we recognized several inherent methodological limitations in this study. Five questions need to be addressed in a future study. First, the selection bias of a retrospective study design represented the main limitation of this study. Our findings remained primarily hypothesis-generating, and going forward, they must be evaluated in the context of randomized evidence, when available. Second, our data lacked detailed information regarding tumor margin status and the intraoperative omental and peritoneal assessment. Because of the surgical codes used, we could not analyze the scope of the performed omentectomy or the mode of surgery, that is whether it was open or minimally invasive. Furthermore, based on the coding schema used by the SEER database, we could not determine whether patients who did not undergo omentectomy received omental biopsies. Third, the database did not contain data regarding the chemotherapy regimen, course, as well as response. Fourth, our analysis focused primarily on CSS without providing any details about local recurrence and distant metastasis after initial treatment. This could be attributed to the unavailability of such details in the SEER database, which could have important implications for studying the impact of adjuvant therapy on this patient population.

## Conclusion

Patients with clinical Stage I epithelial ovarian carcinomas managed by surgery with the inclusion of omentectomy did not have any survival benefit. Thus, routine omentectomy could be potentially omitted when staging EOC patients when grossly omental abnormalities and extra-ovarian disease spread were not identified. The performance of omentectomy should still be a routine practice when tumor spreads beyond the surface of the ovaries. Further, multi-institutional studies, focusing on the incidence of isolated microscopic omental metastases as well as on the oncologic outcomes of patients having a normal-appearing omentum and not undergoing omentectomy, are required to validate our results.

## Data Availability

The raw data supporting the conclusions of this article will be made available by the authors without undue reservation.
