# Supplementary figures and images for: The impact of omentectomy on cause-specific survival of Stage I–IIIA epithelial ovarian cancer: A PSM–IPTW analysis based on the SEER database
Source: Front Surg. 2022 Dec 29;9:1052788. doi: 10.3389/fsurg.2022.1052788 (PMC9836003; doi:10.3389/fsurg.2022.1052788)

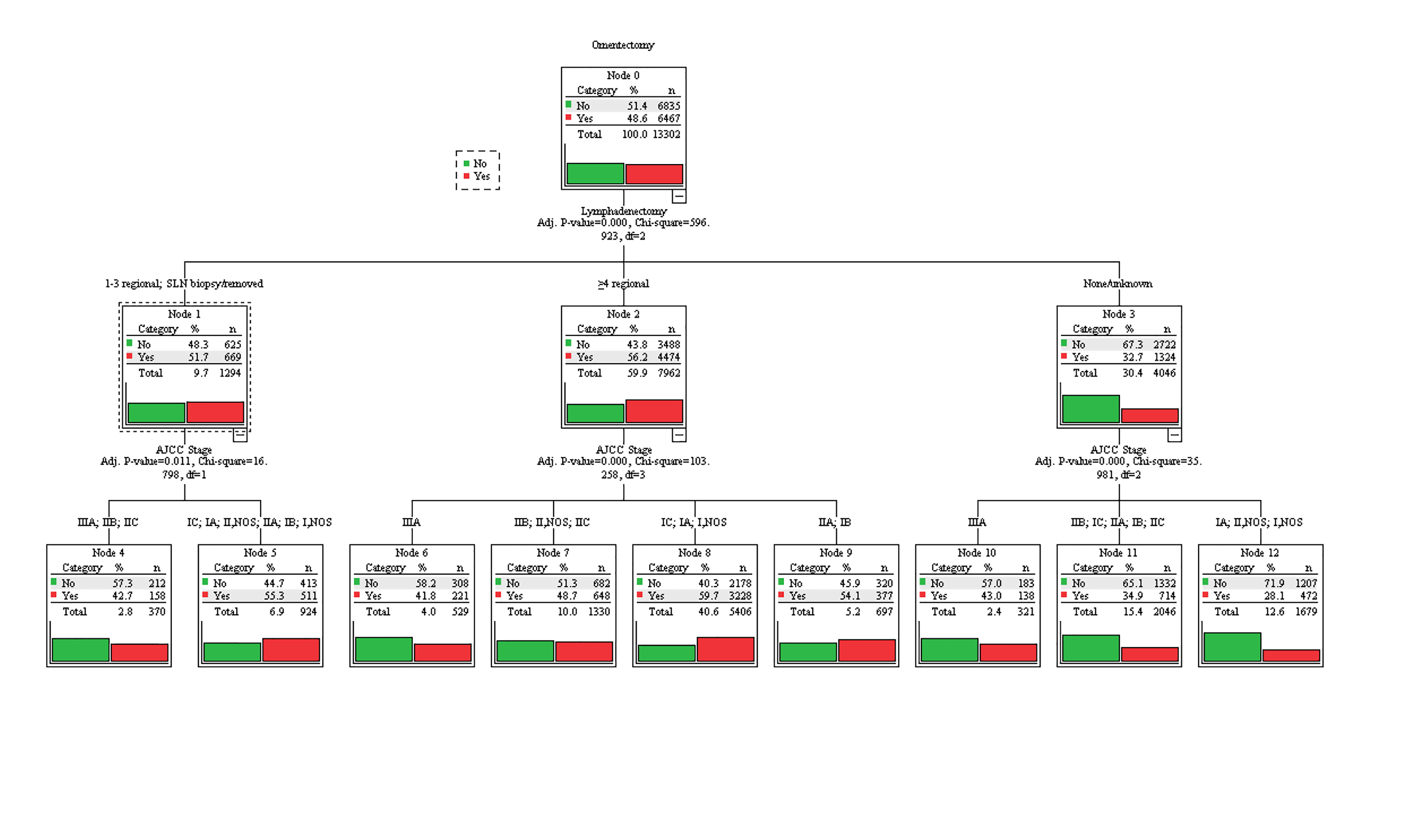

Supplement: Supplementary file 1 [file Image1.tif]
